# Transport through a strongly coupled graphene quantum dot in perpendicular magnetic field

**DOI:** 10.1186/1556-276X-6-253

**Published:** 2011-03-24

**Authors:** Johannes Güttinger, Christoph Stampfer, Tobias Frey, Thomas Ihn, Klaus Ensslin

**Affiliations:** 1 Solid State Physics Laboratory, ETH Zurich, 8093 Zurich, Switzerland; 2 Current Address: JARA-FIT and II, Institute of Physics, RWTH Aachen, 52074 Aachen, Germany

## Abstract

We present transport measurements on a strongly coupled graphene quantum dot in a perpendicular magnetic field. The device consists of an etched single-layer graphene flake with two narrow constrictions separating a 140 nm diameter island from source and drain graphene contacts. Lateral graphene gates are used to electrostatically tune the device. Measurements of Coulomb resonances, including constriction resonances and Coulomb diamonds prove the functionality of the graphene quantum dot with a charging energy of approximately 4.5 meV. We show the evolution of Coulomb resonances as a function of perpendicular magnetic field, which provides indications of the formation of the graphene specific 0th Landau level. Finally, we demonstrate that the complex pattern superimposing the quantum dot energy spectra is due to the formation of additional localized states with increasing magnetic field.

## Introduction

Graphene [1,2], a two-dimensional solid consisting of carbon atoms arranged in a honeycomb lattice has a number of unique electronic properties [3], such as the gapless linear dispersion, and the unique Landau level (LL) spectrum [4,5]. The low atomic weight of carbon and the low nuclear spin concentration, arising from the ≈99% natural abundance of ^12^C, promises weak spin orbit and hyperfine coupling. This makes graphene a promising material for spintronic devices [6,7] and spinqubit based quantum computation [8-11]. Additionaly, the strong suppression of electron backscattering [4,5] makes it interesting for future high mobility nanoelectronic applications in general [12,13]. Advances in fabricating graphene nanostructures have helped to overcome intrinsic difficulties in (i) creating tunneling barriers and (ii) confining electrons in bulk graphene, where transport is dominated by Klein tunneling-related phenomena [14,15]. Along this route, graphene nanoribbons [16-22] and quantum dots [23-30] have been fabricated. Coulomb blockade [23-25], quantum confinement effects [26-28] and charge detection [29] have been reported. Moreover, graphene nanostructures may allow to investigate phenomena related to massless Dirac Fermions in confined dimensions [24,31-36]. In general, the investigation of signatures of graphene-specific properties in quantum dots is of interest to understand the addition spectra, the spin states and dynamics of confined graphene quasi-particles.

Here, we report on tunneling spectroscopy (i.e. transport) measurements on a 140-nm graphene quantum dot with open barriers, which can be tuned by a number of lateral graphene gates [37]. In contrast to the measurements reported in Ref. [27] the more open dot in the present investigation enables us to observe Coulomb peaks with higher conductance and the larger dot size reduces the magnetic field required to see graphene specific signatures in the spectra. We characterize the graphene quantum dot device focusing on the quantum dot Coulomb resonances which can be distinguished from additional resonances present in the graphene tunneling barriers. We discuss the evolution of a number of Coulomb resonances in the vicinity of the charge neutrality point in a perpendicular magnetic field from the low-field regime to the regime where Landau levels are expected to form. In particular, we investigate the device characteristics at elevated perpendicular magnetic fields, where we observe the formation of multiple-dots giving rise to (highly reproducible) complex patterns in the addition spectra.

## Device fabrication

The fabrication process of the presented graphene nano-device is based on the mechanical exfoliation of (natural) graphite by adhesive tapes [24,25,28]. The substrate material consists of highly doped silicon (Si^++^) bulk material covered with 295 nm of silicon oxide (SiO_2_), where thickness (and roughness) of the SiO_2 _top layer is crucial for the Raman [38] and scanning force microscope based identification of single-layer graphene flakes. Standard photolithography followed by metallization and liftoff is used to pattern arrays of reference alignment markers on the substrate which are later used to re-identify the locations of individual graphene flakes on the chip and to align further processing patterns. The graphene flakes are structured to submicron dimensions by electron beam lithography (EBL) and reactive ion etching based techniques to fulfill the nanodevice design requirement. After etching and removing the residual resist, the graphene nanostructures are contacted by an additional EBL step, followed by metallization and lift-off.

**Figure 1:**
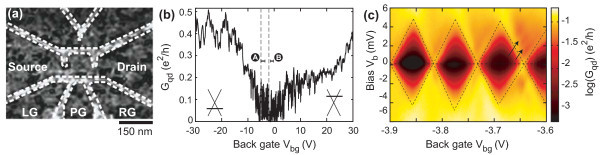
**Device characterization**. **(a) **Scanning force microscopy of the graphene quantum dot device. The overall chemical potential of the device is tuned by a global back gate, where as the right side gate (RG) is used for local asymmetric tuning. The extension of the dot is around 140 nm with 75 nm wide and 25 nm long constrictions. The white dashed lines delineating the quantum dot perimeter are added for clarity. **(b) **Measurement of the source (S)-drain (D) conductance for varying back gate voltage showing a transport gap from around -5 to 3 V (*V*_b _= 200 *μ*V). **(c) **Coulomb diamond measurements in the gap showing a charging energy of around 4.5 meV. This energy is lower than what has been measured in an other dot of similar size (Ref. [26]), most likely because of the increased coupling to the leads. The arrows point to faint lines outside the diamonds. The extracted energy difference of around 1 meV is a reasonable addition energy for excited states. Note that for the measurement in **(c)**, in addition to the BG the right side gate was changed according to *V*_rg _= -0.57·*V*_bg _-1.59 V.

A scanning force microscope image of the final device studied here is shown in Figure 1a. The approximately 140 nm diameter graphene quantum dot is connected to source (S) and drain (D) via two graphene constrictions with a width of ≈75 nm and a length of ≈25 nm, both acting as tunneling barriers. The dot and the leads can be further tuned by the highly doped silicon substrate used as a back gate (BG) and three in-plane graphene gates: the left side gate (LG), the plunger gate (PG) and the right side gate (RG). Apart from the geometry, the main difference of this sample compared to the device presented in Ref. [27] is the higher root mean square variation of the height (*r*_h_) on the island. While there are no visible resist residues on the island of the sample in Ref. [27] with *r*_h _≈ 0.35 nm, there are many dot-like residues on the sample presented here giving *r*_h _≈ 1.1 nm.

## Measurements

All measurements have been performed at a base temperature of *T *= 1.8 K in a variable temperature cryostat. We have measured the two-terminal conductance through the graphene quantum dot device by applying a symmetric DC bias voltage *V*_b _while measuring the source-drain current through the quantum dot with a noise level below 10 fA. For differential conductance measurements a small AC bias, *V*_b,ac _= 100 *μ*V has been superimposed on *V*_b _and the differential conductance has been measured with lock-in techniques at a frequency of 76 Hz.

In Figure 1b we show the conductance *G*_qd _as a function of back gate voltage at low bias (*V*_b _= 200 *μ*V) highlighting the strong suppression of the conductance around the charge neutrality point (-5 <*V*_bg _< 3 V) due to the so-called transport gap [19-22]. Here we tune transport from the hole to the electron regime, as illustrated by the left and the right inset in Figure 1b. The large number of resonances with amplitudes in the range of up to 0.1 *e*^2^/*h *inside the gap region may be due to both, (i) resonances in the graphene constrictions acting as tunneling barriers [4] (and thus being mainly responsible for the large extension of this transport gap) and (ii) Coulomb resonances of the quantum dot itself (see also examples of Coulomb diamonds in Figure 1c). At room temperature these resonances disappear and a conductance value of 0.76 *e*^2^/*h *is measured at *V*_bg _= 0 V.

## Coulomb blockade measurements at *B *= 0 T

**Figure 2:**
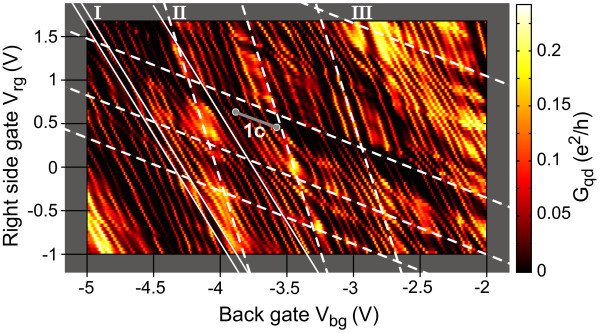
**Conductance of the quantum dot with varying right gate and back gate voltage measured at bias voltage *V*_b _= 200 *μ*V**. Coulomb resonances and modulations of their amplitude with different slopes are observed (dashed white lines). The extracted relative side gate back gate lever arms are ,  and . Lever arm (III) is attributed to resonances in the right constriction which are strongly tuned by the right side gate. In contrast resonances with lever arm (II) are only weakly affected by the right side gate and therefore attributed to states in the left constriction. The periodic resonances marked with (I) are attributed to resonances in the dot in agreement with the intermediate slope.

By focusing on a smaller back gate voltage range within the transport gap (indicated by the dashed lines in Figure 1b) and measuring the conductance as a function of *V*_bg _and the right side gate *V*_rg _much more fine-structure appears, as shown in Figure 2. A large number of resonances is observed with sequences of diagonal lines (see white lines in Figure 2) with different slopes, corresponding to different lever arms (*α*'s). By sweeping the right side gate (*V*_rg_) we break the left-right symmetry of the transport response (see also Figure 1a). This allows us to distinguish between resonances located either near the quantum dot or the left and right constriction. The steeper the slope in Figure 2 the less this resonance can be electrostatically tuned by the right side gate and, consequently, the larger the distance between the corresponding localized state and the right side gate. Subsequently, the steepest slope (II, corresponding to ) can be attributed to resonances in the left constriction and the least steepest slope (III, ) belongs to resonances in the right constriction. Both are highlighted as white dashed lines in Figure 2. The Coulomb resonances of the quantum dot appear with an intermediate slope (I, ) and exhibit clearly the smallest spacing in back gate voltage, Δ*V*_bg _≈ 0.1 V. This is a good indication that they belong to the largest charged island in the system, which obviously is the 140 nm large graphene quantum dot, which is much larger than the localized states inside the graphene constrictions acting as tunneling barriers.

Corresponding Coulomb diamond measurements [39], that is, measurements of the differential conductance as a function of bias voltage *V*_b _and *V*_bg _(i.e. *V*_rg _= -0.57·*V*_bg _- 1.59 V) have been performed along the (diagonal) solid gray line in Figure 2 and are shown in Figure 1c. From the extent of these diamonds in bias direction we estimate the average charging energy of the graphene quantum dot to be *E*_c _= 4.5 meV, which is in reasonable agreement with the size of the graphene quantum dot [23,25,26]. Moreover, we observe faint strongly broadened lines outside the diamonds running parallel to their edges, as indicated by arrows in Figure 1c. The extracted energy difference of roughly 1 meV is reasonable for electronic excited states in this system [26].

## Coulomb resonances as a function of a perpendicular magnetic field

**Figure 3:**
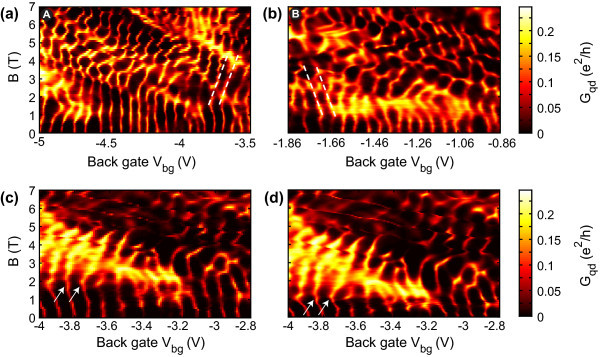
**Evolution of Coulomb peaks under the influence of a magnetic field in different gate voltage regimes (*V*_b _= 200 *μ*V)**. **(a) **More on the hole side. **(b) **More on the electron side. In contrast to **(a) ***V*_rg _= -2.15 V is applied to the right gate in **(b)**. The effect of the right gate to the dot is taken into account in the back gate scale to allow comparison with Figure 1b. **(c**, **d) **Reproducibility of the measurement for different magnetic field sweep directions (0-7 T in **(c)**, 7-0 T in **(d)**). The right side gate is changed according to *V*_rg _= -0.57·*V*_bg _- 1.59 V (see Figure 2), with an applied bias of *V*_b _= 200 *μ*V.

In Figure 3 we show a large number of Coulomb resonances as function of a magnetic field perpendicular to the graphene sample plane. The measurement shown in Figure 3a has been taken in the back gate voltage range from *V*_bg _= -5 to -3.5 V, at *V*_rg _= 0 V (highlighted by the horizontal line (A) in Figure 1b). Thus we are in a regime where transport is dominated by holes (i.e. we are at the left hand side of the charge neutrality point in Figure 1b), which is also confirmed by the evolution of the Coulomb resonances in the perpendicular magnetic field as shown in Figure 3a. There is a common trend of the resonances to bend towards higher energies (higher *V*_bg_) for increasing magnetic field, in good agreement with Refs. [27,28,32-34]. The finite magnetic field introduces an additional length scale  which competes with the diameter *d *of the dot. Therefore, the ratio *d*/ℓ_B _is a relevant parameter for the observation of Landau levels in graphene quantum dot devices. Here, the comparatively large size (*d *≈ 140 nm) of the dot promises an increased spectroscopy window for studying the onset and the formation of Landau levels in graphene quantum dots in contrast to earlier work [27,28] (where *d *≈ 50 nm). Moreover, we expect that in larger graphene quantum dots, where the surface-to-boundary ratio increases edge effects should be less relevant. In Figure 3a, c, d we indeed observe some characteristics of the Fock-Darwin-like spectrum [32-34] of hole states in a graphene quantum dot in the near vicinity of the charge neutrality point: (i) the levels stay more or less at constant energy (gate voltage) up to a certain *B*-field, where (ii) the levels feature a kink, whose *B*-field onset increases for increasing number of particles, and (iii) we observe that the levels convergence towards higher energies (see white dashed lines in Figure 3a). The pronounced kink feature (see arrows in Figure 3c, d) indicate filling factor *ν *= 2 in the quantum dot. However, this overall pattern is heavily disturbed by additional resonances caused by localized states, regions of multi-dot behavior, strong amplitude modulations due to constriction resonances and a large number of additional crossings, which are not yet fully understood. This becomes even worse when investigating the electron regime (see horizontal line (B) in Figure 1b), as shown in Figure 3b. Individual Coulomb resonances can (only) be identified for low magnetic fields *B *< 2 T and a slight tendency for their bending towards lower energies might be identified (please see white dashed lines in Figure 3b). For magnetic fields larger than 3 T it becomes very hard to identify individual Coulomb resonances in the complex and reproducible conductance pattern.

In order to demonstrate the reproducibility of these complex patterns we show an up (Figure 3c) and a down (Figure 3d) sweep of the very same *B *- *V*_bg _parameter space. These two measurements, have different resolution and thus different sweep rates in both the *B *and *V*_bg _direction. However, all the individual features are highly reproducible (but hard to understand) despite the fact that we find some small hysteresis in magnetic field for *B *< 3 T (see white arrows in Figure 3c, d). The origin of the complex patterns shown in Figure 3 can be understood when having a closer look at charge stability diagrams (such as Figure 2) for different magnetic fields.

**Figure 4:**
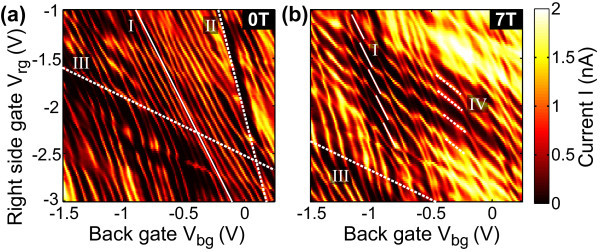
**Dot conductance as a function of right gate and back gate voltage at a magnetic field of (a) 0 T and (b) 7 T**. The spectrum is dominated by dot resonances marked with the solid line in **(a) **with a relative lever arm of  (see also Figure 2). **(b) **At a magnetic field of 7 T a hexagon pattern with two characteristic slopes is observed. Their corresponding lever arms are  attributed to the dot and  origin around the right constriction.

In Figure 4a we show an example of a sequence of dot Coulomb resonances in the *V*_rg_-*V*_bg _plane. The slope corresponding to  and the spacing of Δ*V*_bg _≈ 0.1 V are in good agreement with Figure 2, and lead to the conclusion that we observe single quantum dot behaviour over a large parameter range. However, if we measure the current in the very same *V*_rg _- *V*_bg _parameter space at *B *= 7 T the pattern changes significantly and the diagonal lines are substituted by a strong hexagonal pattern (see dashed lines) typical for two coupled quantum dots [40]. The two states forming the hexagon pattern show relative lever arms of  and . While the resonances with  are attributed to the original dot,  corresponds to a new and strongly coupled localization formed close to the right constriction. Additional resonances from the right constriction with  (see above) are still visible.

We interpret the magnetic field dependence in the following way. At low but increasing magnetic field we see in almost all measurements an increase of the conductance through the dot (see, e.g. Figure 3). Assuming diffusive boundary scattering such a conductance onset in magnetic field occurs due to reduced backscattering [41] and has been observed in other measurements on graphene nanoribbons [42,43]. The maximum conductance is reached around *B *≈ 1.5 T corresponding to a magnetic length  nm in rough agreement with the size of the constrictions. As the magnetic field is further increased the complex pattern with many crossings starts to emerge, attributed to the formation of additional quantum dots around the right constriction with strong coupling to the original dot. The formation of such localized puddles is understood as a consequence of the increased magnetic confinement where ℓ_B _is getting smaller than the extension of potential valleys induced by disorder.

## Conclusion

In summary, we have presented detailed studies of transport through an open and larger graphene quantum dot (compared to Ref. [27]) in the vicinity of the charge neutrality point as a function of perpendicular magnetic field. The evolution of Coulomb resonances in a magnetic field showed the signatures of Landau level formation in the quantum dot. Indications for the crossing of filling factor *ν *= 2 are obtained by the observation of kinks in spectral lines before bending towards the charge neutrality point. However, the observation is disturbed by the formation of a pronounced additional localized state at high magnetic fields in the vicinity of the right constriction. Although the use of open constrictions enhances the visibility of the Coulomb peaks and reduces the transport-gap region, emerging pronounced parasitic localized states make the analysis very difficult. For a further in-depth analysis of the addition spectra around the electron-hole crossover, it is hence beneficial to minimize the amount of disorder and to use clearly defined constrictions. These should be thin compared to the dot diameter to get different energy scales for quantum dot resonances and constriction resonances, which are easy to distinguish. However, the constrictions need to be wide enough to enable conductance measurements around the electron-hole crossover without a charge detector.

## Abbreviations

BG: back gate; EBL: electron beam lithography; LL: Landau level; LG: left side gate; PG: plunger gate; RG: right side gate; SiO_2_: silicon dioxide.

## Competing interests

The authors declare that they have no competing interests.

## Authors' contributions

KE, TI, CS and JG designed the experiment. JG fabricated the sample. TF and JG carried out the transport measurements. All authors analyzed the measurements. JG and CS wrote the paper. All authors read and approved the final manuscript.
